# Simulation‐Based Learning in Oral Radiology: Students’ Perceptions of Training in Intraoral Radiographic Techniques Across Two Dental Institutes

**DOI:** 10.1002/jdd.70138

**Published:** 2025-12-30

**Authors:** Abeer A. Almashraqi, Ankur Jethlia, Rebecca Glanville, Hanin Daas, Kamran Ali

**Affiliations:** ^1^ College of Dental Medicine QU Health, Qatar University Doha Qatar; ^2^ College of Dentistry Jazan University Jazan Saudi Arabia; ^3^ Faculty of Health Plymouth University Plymouth UK

**Keywords:** intraoral radiographs, mannequin, oral radiology, qualitative analysis, students, thematic analysis

## Abstract

**Objective:**

Simulation‐based learning (SBL) in oral radiology offers a safe, structured environment that supports students’ transition to clinical practice. However, limited research has captured students' perceptions of SBL for intraoral radiography training. This study aimed to explore the perceptions of dental students about SBL in intraoral radiography using a mixed‐methods approach across two dental institutions.

**Methods:**

Third‐year undergraduate dental students from two institutions were recruited. All students were trained on both periapical (paralleling technique in Institution A and bisecting angle technique in Institution B) and bitewing radiographs using a mannequin. Data were collected through a 9‐item Likert‐scale survey and three open‐ended questions. Quantitative data were analyzed using descriptive statistics and Mann–Whitney *U* test, while qualitative data underwent thematic analysis.

**Results:**

A total of 44 students expressed overall positive perceptions of SBL, with a mean score of 4.27. The highest‐rated item was “Readiness for clinical transition” (mean = 4.53), and the lowest was “Realism” (mean = 3.81). Institution A students showed significantly more positive responses than those from Institution B in items: develop self‐confidence, skill development, and skill mastery through repetition. Thematic analysis supported quantitative findings and revealed four key themes. Students valued the role of SBL in skill development, providing a psychologically safe learning environment, and bridging theory and practice. Students noted barriers about mannequin realism, technical challenges, and access issues. They also provided suggestions for improving the SBL experience.

**Conclusion:**

The findings indicate that students value SBL as an effective approach for teaching core oral radiology skills and preparing students for clinical training. Enhancing mannequin design and practice opportunities may further improve undergraduate learning experiences in simulated settings.

## 1 Introduction

A competency‐based approach is well‐established in contemporary dental education, and graduating dental students are expected to be competent at performing a range of clinical dental procedures [1, 2, 3]. A competency‐based approach aims to equip undergraduate dental students with a broad range of clinical skills in operative procedures and ultimately protect the patients and the public. Clinical education is a rapidly evolving field and is witnessing the increasing use of technology and the development of new training models, especially with greater utilization of simulated dental learning environments [4]. Simulation‐based learning (SBL) refers to a structured educational approach that uses simulated environments to allow students to practice procedures safely, receive feedback, and build confidence before working with real patients. This offers a safe learning space for students and facilitates a smooth transition into clinical practice [5].

Oral radiography and radiographic reporting are an integral component of clinical dentistry and provide the foundation for diagnosing various oral and dental disorders. The precise stage at which oral radiography teaching is introduced in undergraduate dental curricula varies with the curriculum structure of individual dental institutions. Given that radiography is a core requirement for accurate diagnosis and treatment planning, there is merit in providing essential training at an early stage.

Traditionally, diagnostic radiology education has been delivered in workplace‐based settings. The undergraduate dental students received didactic teaching in oral radiology, followed by clinical placements under the supervision of a radiologist and/or dental practitioners to learn and consolidate their radiography skills on real patients. Oral radiology involves the production of harmful biological radiations, that is, X‐rays, and there is a higher risk to patients and clinical staff if novice learners are allowed to expose radiographs without structured training and assessments. Training directly in clinical settings can be challenging for the students and adversely impact their confidence, and also potentially put patients’ safety at risk [6]. Like with many other dental disciplines, oral radiography training in a simulated environment appears to be an appropriate approach to support student learning and ensure safety in clinical settings [7, 8]. There is a growing recognition of the benefit of simulated learning environments, and many institutions, including our own, are developing structured preclinical training in oral radiography. Simulated environment offers several advantages, including a safe environment for familiarization with radiography equipment and software, repeated opportunities to consolidate basic skills in oral radiography without any concerns regarding patient safety, and standardized assessments. Despite its advantages, some limitations have been raised, such as cost, realism, accessibility, and technical issues.

Previous studies have explored the impact of virtual reality to improve students’ skills in assessing and interpreting radiographs in medical radiology [9, 10, 11, 12]. To our knowledge, limited studies have explored the effectiveness of SBL in oral radiology teaching [13, 14, 15, 16]. These studies focused on the students’ performance scores in radiographic interpretation and reported the SBL as an effective educational tool. Some of these studies focused on the SBL using virtual reality and concluded the effectiveness of SBL approaches in improving radiographic technique proficiency [15, 16]. However, no previous research has delved into capturing the perspectives of students regarding SBL in oral radiography, specifically the training of taking radiographs. Like any educational method, which has its advantages and limitations, it is essential to study the SBL from the students’ perspectives. Therefore, this study aimed to comprehensively explore students' perceptions of SBL for training in intraoral radiographic techniques, using a mixed‐methods approach across two institutions.

### 1.1 Conceptual Framework

With the current advancement in dental and medical education, there is an emphasis on social and experiential approaches to learning. These concepts focus on the fact that the learner learns in a social context where the learner starts from the zone of proximal development and is then immersed in their communities of practice, as suggested by Vygotsky [17] and Lave and Wenger [18], respectively. Simultaneously, they will benefit from learning by performing particular tasks in a specific context during their training, initially by observing and performing, and then reflecting and adopting them [19].

Vygotsky's concept of the *zone of proximal development* underpins the structured supervision provided during mannequin‐based training. In this framework, learning occurs most effectively when students engage in tasks that they cannot yet accomplish independently but can achieve with appropriate guidance and feedback from instructors. Within the simulation sessions, tutors scaffold students’ learning by offering step‐by‐step demonstrations, corrective feedback, and opportunities for repeated practice, thereby facilitating the gradual transition from assisted to autonomous performance.

Simulation‐based education, in general, and oral radiology, in particular, depend on applying both experiential and situated learning theories suggested by Kolb [19] and Lave and Wenger [20]. Learning in a simulated environment allows the students to learn by observing, performing, linking the new knowledge with the existing experience, and using tutor feedback and self‐reflection to improve their performance. In addition, the students practice in a social context with their communities of practice, where they learn from each other. The simulation oral radiology lab functioned as such a community, where students actively collaborated, observed peers, exchanged feedback, and collectively refined their radiographic skills. This social interaction and shared engagement not only promoted technical competence but also nurtured professional communication and confidence essential for real clinical environments. These approaches allow students to acquire the needed clinical skills, basic knowledge, and clinical reasoning skills by observing, doing, making mistakes, reflecting, repeating actions, and, most importantly, learning safely and ensuring a smooth transition to clinical settings [21, 22].

## 2 Materials and Methods

### 2.1 Research Ethics

The study's ethical approval has been obtained from the Institutional Review Boards at Qatar University and the Standing Committee for Scientific Research at Jazan University with the reference numbers “QU‐IRB 1652‐EA/22” and “CODJU‐2213F,” respectively.

The data collection process was anonymous, and the participants' responses could not be tracked or identified. The aim of the study was explained to the participants, and they were informed that their participation was completely voluntary and that they would not be disadvantaged if they decided not to participate or declined to take part in the study at any stage. Then, their consent was taken at the beginning of the electronic survey to confirm they agreed to participate.

### 2.2 Study Design and Settings

A cross‐sectional study following a structured educational intervention was conducted to explore the students' perception of the SBL approach in oral radiology using a mixed‐methods approach that was conducted at the College of Dental Medicine, Qatar University, and the Faculty of Dentistry, Jazan University.

### 2.3 Sampling Technique and Participants

Purposive sampling was used to include third‐year students of the undergraduate dental programs at the two institutions. Potential participants were invited to participate in the research by email, accompanied by a participant information sheet with details of the scope, purpose of the study, and contact details of the research team.

### 2.4 Eligibility Criteria

Undergraduate dental students who were at least 18 years old and in their third year of study were recruited. Students who had withdrawn from education or declined to participate in the study were excluded from the study. The recruitment process was extended over one academic year to include one cohort of students from both institutions.

### 2.5 Research Instrument

A questionnaire consisting of nine closed‐ and three open‐ended items was developed by the research team, composed of two experienced clinical dental academics (A.A. and K.A.). The questionnaire focused on the students’ perception of their learning experiences in oral radiology within a simulated setting from different perspectives, as presented in Table 1. The face and content validity of the items was determined through a detailed mapping of the questionnaire items with the learning outcomes of intraoral radiography described by professional organizations [1, 3, 23]. In addition, the internal consistency of the questionnaire was assessed using Cronbach's alpha (*α* = 0.87), confirming high reliability of the instrument.

**TABLE 1:** Student responses to simulation‐based learning questionnaire across institutions A and B.

Survey items	Text	Institution	Strongly agree	Agree	Neutral	Disagree	Strongly disagree
1‐Develop self‐confidence	Training on the mannequin improved my confidence in learning oral radiology	A	10 (71.43%)	4 (28.57%)	0	0	0
		B	8 (26.67%)	17 (56.67%)	5 (16.67%)	0	0
		Combined	18 (40.91%)	21 (47.73%)	5 (11.36%)	0	0
2‐Translating radiographic concepts into practice	Mannequin training facilitated the accurate geometric application of each intraoral radiographic projection	A	5 (35.71%)	5 (35.71%)	4 (28.57%)	0	0
		B	9 (30.00%)	15 (50.00%)	6 (20.00%	0	0
		Combined	14 (31.82%)	20 (45.45%)	10 (22.73%)	0	0
3‐Realism	Radiographs of the teeth and supporting structures on the mannequins accurately simulated real structures	A	3 (21.43%)	8 (57.14%)	2 (14.29%)	1 (7.14%)	0
		B	8 (26.67%)	10 (33.33%)	8 (26.67%)	3 (10.00%)	1 (3.33%)
		Combined	11 (25.00%)	18 (40.91%)	10 (22.73%)	4 (9.09%)	1 (2.27%)
4‐Skill development	Training on the mannequin improved my fine motor skills	A	9 (64.29%)	5 (35.71%)	0	0	0
		B	7 (23.33%)	15 (50.00%)	7 (23.33%)	1 (3.33%)	0
		Combined	16 (36.36%)	20 (45.45%)	7 (15.91%)	1 (2.27%)	0
5‐Skill mastery through repetition	Repeated practice on a mannequin is helpful in improving learner performance	A	12 (85.71%)	2 (14.29%)	0	0	0
		B	12 (40.00%)	15 (50.00%)	3 (10.00%)	0	0
		Combined	24 (54.55%)	17 (38.64%)	3 (6.82%)	0	0
6‐Risk‐free learning environment	Training on a mannequin can help improve patient safety	A	8 (57.14%)	5 (35.71%)	1 (7.14%)	0	0
		B	12 (40.00%)	13 (43.33%)	4 (13.33%)	1 (3.33%)	0
		Combined	20 (45.45%)	18 (40.91%)	5 (11.36%)	1 (2.27%)	0
7‐Readiness for clinical transition	Assessment of radiography skills of students on a mannequin should precede clinical training	A	10 (71.43%)	4 (28.57%)	0	0	0
		B	16 (53.33%)	10 (33.33%)	3 (10.00%)	0	0
		Combined	26 (59.09%)	14 (31.82%)	3 (6.82%)	0	0
8‐Appropriateness in dental education	A simulated environment is appropriate for learning radiography skills	A	5 (35.71%)	9 (64.29%)	0	0	0
		B	14 (46.67%)	15 (50.00%)	1 (3.33%)	0	0
		Combined	19 (43.18%)	24 (54.55%)	1 (2.27%)	0	0
9‐Standardization and curriculum integration	Training on a mannequin should be used as a standard for pre‐clinical education in radiology	A	9 (64.29%)	5 (35.71%)	0	0	0
		B	13 (43.33%)	13 (43.33%)	3 (10.00%)	1 (3.33%)	0
		Combined	22 (50.00%)	18 (40.91%)	3 (6.82%)	1 (2.27%)	0
10‐Overall perception		A	56.35%	37.30%	5.56%	0.79%	0.00%
		B	36.67%	45.55%	14.81%	2.22%	0.37%
		Combined	42.93%	42.93%	11.87%	1.77%	0.25%

The development of the questionnaire was aimed at eliminating biases related to person factors. In this regard, the following strategies were adopted:

- Recall of relevant skills and behaviors was easy for the respondents.
- The items were worded unambiguously to facilitate the interpretation of items by the participants.
- Online administration of the scale allowed blind reporting by the participants so that they felt confident to provide their responses. This also precluded any influence of the researcher on the responses provided by the participants.

Having generated the potential items for inclusion in the questionnaire, pretesting of items was conducted in line with established practices. Undergraduate dental students (*N* = 5) and dental academics (*N* = 5) participated in pretesting of the questionnaire. The purposes of pretesting scale items were as follows:

1. The scale items are comprehensible and unambiguous.
2. Scoring categories can be interpreted with ease.
3. Determine the content and face validity of scale items.

Minor amendments were made to the questionnaire to enhance the clarity of the language. The final questionnaire consisted of nine closed‐ended items scored on a 5‐point Likert scale with the responses assigned a numerical code (*strongly agree*: 5, *agree*: 4, *neutral*: 3, *disagree*: 2, and *strongly disagree*: 1). Three open‐ended items were also included in the questionnaire, which included the advantages and limitations of SBL in oral radiology as well as suggestions for improvement.

### 2.6 Data Collection

**FIGURE 1:**
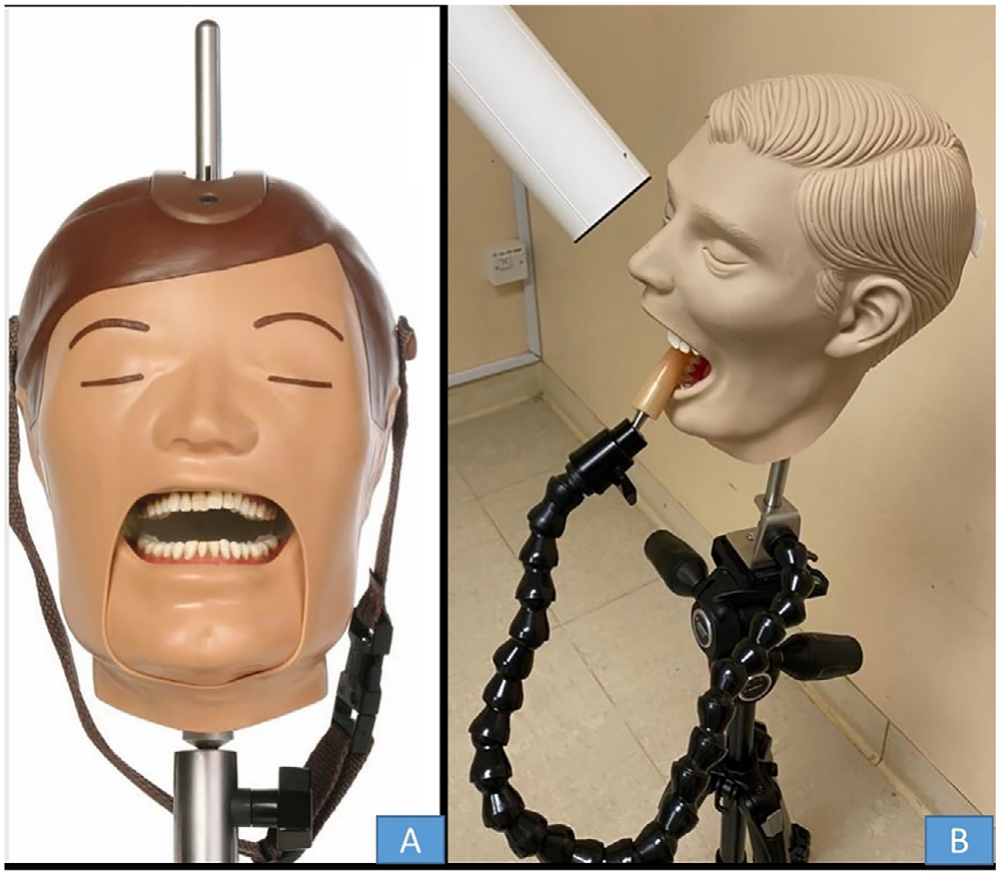
Mannequin used in (A) Institution A, Dentsply Rinn Dxttr and (B) Institution B, tripod x‐ray manikin.

Teaching and training of the participants on intraoral radiographs (periapical and bitewing) was carried out at both institutions for one semester by the research team (A.A. and A.J.). The participants at Institution A were trained for both periapical and bitewing radiographs using the paralleling technique and the receptor holders, respectively, on the Dentsply Rinn Dxttr, Sirona mannequin (Figure 1A). Participants at Institution B received training on the bisecting angle technique using finger support for periapical and bitewing tapes for bitewing radiographs on the Tripod x‐ray Manikin set, Nissin (Figure 1B). The mannequins used in both institutions have almost the same fidelity.

Each cohort completed one session (2 h) a week of simulation sessions for 14 weeks, covering anterior, canine, and posterior region periapical and posterior horizontal bitewing radiographs under instructor supervision. Each session began with a faculty demonstration of patient positioning, receptor placement, use of receptor holders or finger support, and radiation and infection control safety practices, including operator positioning behind protective barriers. Then, the students practiced in groups during which they learned from each other, reflected on their work, and provided feedback. The participants were invited to complete an online questionnaire using Google Forms. Prior to accessing the questionnaire, each participant was required to sign an electronic consent form to confirm they understood the purpose of the study, their participation was voluntary, and the data would be processed anonymously.

### 2.7 Data Analyses

Descriptive statistics, including confidence intervals, were calculated for each institution and the combined dataset. A Mann–Whitney *U* test was used to determine any significant variation between the institutions and the gender results. All data were analyzed and visualized using the Statistical Package for Social Sciences (SPSS) software, Version 21 (Armonk, New York: IBM Corp.).

The analysis of the open‐ended questions for the two institutions was conducted independently by two researchers (A.A. and K.A.) to ensure trustworthiness. The process began with data familiarization, where students’ responses were thoroughly reviewed multiple times to identify initial patterns and insights. Meaningful units of data were systematically coded, allowing for the identification of recurring ideas and perspectives. These codes were then clustered into broader themes, which were refined through iterative reviews to ensure they accurately captured participants' views and aligned with the overall dataset. The researchers compared their codes for consistency and resolved any discrepancies through discussion. Finally, the themes were synthesized into a cohesive narrative that was directly linked to the research objectives, providing a comprehensive understanding of the students’ perception of SBL in oral radiology. Direct participant quotes were included throughout the analysis to ground the findings in the data and enhance authenticity.

## 3 Results

A total of 44 dental students responded to the questionnaire, with 14 from Institution A and 30 from Institution B, yielding a response rate of 100% at both participating institutions.

### 3.1 Quantitative Analysis

A count of each response by the institution and overall is provided in Table 1, which shows that most of the answers were between strongly agree and agree, with the overall percentages of all questions of both schools as 85.86%.

The combined results of both institutions are presented in Table 2, with a mean of 4.27 for the overall perception. The results were positive overall, with the highest score of 4.53 related to the survey Item 7 “Readiness for clinical transition,” followed by Items 5, 8, and 9 “skill mastery through repetition, appropriateness in dental education, and standardization and curriculum integration” with the mean of 4.49, 4.42, and 4.40, respectively. However, Item 3, “realism,” received the lowest scores overall (mean of 3.81), followed by Item 2, “translating radiographic concepts into practice” (mean of 4.09).

**TABLE 2:** Descriptive and rank‐based statistical comparison of students’ perceptions of simulation‐based learning in oral radiology, stratified by institution.

Survey items	Institution	Mean	Std. deviation	95% CI lower	95% CI upper	Mean rank	*p* value
1‐Develop self‐confidence	A	4.71	0.469	4.44	4.98	29.93	0.004^*^
	B	4.10	0.662	3.85	4.36	19.03	
	Combined	4.30	0.674	4.09	4.51	—	—
2‐Translating radiographic concepts into practice	A	4.07	0.829	3.59	4.55	22.29	0.935
	B	4.10	0.712	3.83	4.38	22.60	
	Combined	4.09	0.750	3.86	4.32	—	—
3‐Realism	A	3.93	0.829	3.45	4.41	24.11	0.551
	B	3.76	1.088	3.36	4.16	21.75	
	Combined	3.81	0.982	3.51	4.12	—	—
4‐Skill development	A	4.64	0.497	4.36	4.93	30.07	0.004^*^
	B	3.93	0.785	3.63	4.23	18.97	
	Combined	4.16	0.785	3.92	4.40	—	—
5‐Skill mastery through repetition	A	4.86	0.363	4.65	5.07	29.57	0.005^*^
	B	4.30	0.651	4.06	4.56	19.20	
	Combined	4.49	0.631	4.29	4.68	—	—
6‐Risk‐free learning environment	A	4.50	0.650	4.12	4.88	25.54	0.242
	B	4.21	0.805	3.90	4.52	21.08	
	Combined	4.30	0.773	4.06	4.54	—	—
7‐Readiness for clinical transition	A	4.71	0.469	4.44	4.98	24.79	0.241
	B	4.45	0.686	4.19	4.71	20.66	
	Combined	4.53	0.631	4.34	4.73	—	—
8‐Appropriateness in dental education	A	4.36	0.497	4.07	4.64	21.18	0.592
	B	4.45	0.568	4.23	4.67	23.12	
	Combined	4.42	0.545	4.25	4.59	—	—
9‐Standardization and curriculum integration	A	4.64	0.497	4.36	4.93	26.36	0.130
	B	4.28	0.785	3.97	4.58	20.70	
	Combined	4.40	0.728	4.17	4.62	—	—
10‐Overall perception	A	4.49	0.340	4.30	4.69	25.10	0.186
	B	4.16	0.496	3.99	4.37	21.16	
	Combined	4.27	0.760	4.03	4.50	—	—

*Note*: Responses were rated on a 5‐point Likert scale (1 = *strongly disagree*, 5 = *strongly agree*).

Abbreviations: —, values not applicable for combined group comparisons; CI, confidence intervals.

* *p* values are based on the Mann–Whitney *U* test.

Regarding the comparison of the two institutions, Institution B appeared less optimistic about the mannequin radiography teaching than Institution A. The differences between the institutions were statistically significant in items “develop self‐confidence, skill development, and skill mastery through repetition” (*p* ≤ 0.005; Table 2). In contrast, no statistically significant differences were observed in the students’ perception when compared by gender (Table 3).

**TABLE 3:** Descriptive and rank‐based statistical comparison of students’ perceptions of simulation‐based learning in oral radiology, stratified by gender.

Survey items	Gender	Mean	SD	95% CI lower	95% CI upper	Mean rank	*p* value
1‐Develop self‐confidence	Female	4.39	0.63	4.18	4.64	23.84	0.287
	Male	4.13	0.74	3.72	4.54	19.90	
2‐Translating radiographic concepts into practice	Female	4.04	0.74	3.79	4.29	21.57	0.472
	Male	4.20	0.78	3.77	4.63	24.30	
3‐Realism	Female	3.75	0.89	3.46	4.07	20.98	0.252
	Male	3.93	1.16	3.29	4.58	25.43	
4‐Skill development	Female	4.21	0.74	3.93	4.46	23.00	0.698
	Male	4.07	0.88	3.58	4.56	21.53	
5‐Skill mastery through repetition	Female	4.46	0.64	4.25	4.68	21.91	0.634
	Male	4.53	0.64	4.18	4.89	23.63	
6‐Risk‐free learning environment	Female	4.18	0.72	3.93	4.43	20.07	0.056
	Male	4.53	0.83	4.07	5.00	27.20	
7‐Readiness for clinical transition	Female	4.57	0.63	4.36	4.79	22.75	0.535
	Male	4.47	0.64	4.11	4.82	20.60	
8‐Appropriateness in dental education	Female	4.32	0.55	4.11	4.54	20.48	0.096
	Male	4.60	0.51	4.32	4.88	26.40	
9‐Standardization and curriculum integration	Female	4.29	0.76	4.00	4.54	20.62	0.133
	Male	4.60	0.63	4.25	4.95	26.13	
10‐Overall perception	Female	4.27	0.474	4.12	4.41	21.53	0.486
	Male	4.34	0.487	4.07	4.61	24.37	

*Note*: Responses were rated on a 5‐point Likert scale (1 = *strongly disagree*, 5 = *strongly agree*). *p*‐values are based on the Mann–Whitney *U* test.

Abbreviation: CI, confidence intervals.

### 3.2 Thematic Analysis of Open‐Ended Questions

The key themes from qualitative data analysis are summarized below.

#### 3.2.1 Theme 1: Enhanced Skill Development and Safe Learning Environment

Students widely appreciated how simulated training enhanced their practical skill development, clinical preparedness, and confidence without putting real patients at risk. They also valued the psychologically safe learning environment created by SBL.

“To get the feel of how it would be taking radiographs before actually working on patients. To practice until we are perfect, then move on to real patients.” Institution A

“We can make mistakes without the risk of overexposing the patient.” Institution A

“Improve safety for the students and patients.” Institution B

“Learning by practice is helpful to simulate the same environment of the clinic.” Institution B

“Improvement of students' motor skills for proper dealing with patients.” Institution B

#### 3.2.2 Theme 2: Bridging Theory With Practice

Students praised the simulation experience for reinforcing theoretical learning and preparing them for clinical application. They highlighted how simulation acts as a bridge, helping students make sense of complex theoretical content through hands‐on repetition and practice.

“Applying the theoretical part on situations close to reality.” Institution A

“Knowing the position of the receptor, holder, and x‐ray tube.” Institution A

“It's a perfect method for applying the theoretical information in a pre‐clinical environment that will help make the clinical practice much easier.” Institution A

“It helps in making the students more experienced in radiographic geometry.” Institution B

#### 3.2.3 Theme 3: Barriers

Despite its advantages, many students emphasized that mannequins do not fully replicate human anatomy or patient behavior, limiting the realism of their learning experience compared to real‐patient experience.

“It doesn't reflect the varieties of anatomy of each patient.” Institution A

“Some patient factors are not applicable in mannequins, like patient movement and positioning.” Institution A

“The reality of touch is missing, but still excellent.” Institution B

“Do not respond or feel—I can hurt it without realizing that I might hurt a real patient.” Institution B

“Too perfect—real patients will be a completely different experience since not all people have the same occlusion and teeth.” Institution A

In addition, students encountered technical and operational issues, such as mechanical rigidity, unstable bite blocks, loose parts, and unrealistic positioning. It is worth mentioning that technical issues were different in each institute according to the type of mannequin used. These difficulties affected their ability to perform radiographs correctly.

“Closing the mouth of the mannequin and the overlapping as a result of crowding.” Institution A

“Sometimes we can't adjust the holder properly because of the bite of the mannequin.” Institution A

“The finger support wasn't a good stabilizer.” Institution B

“Intraoral bisecting angle technique—moving the mannequin's finger to stabilize the film is so difficult sometimes.” Institution B

“Sometimes teeth fall out and get lost.” Institution B

Moreover, students expressed concerns about the number of available mannequins and access time to simulation labs. Insufficient resources limited students' opportunities for repetition, a key principle in mastering technical procedures.

“Stress due to only one time per week to train—not available at all times.” Institution B

“The quality of the mannequin wasn't the best, and the amount of them available is short.” Institution A

“Difficulty with placing the film in the mouth, and limited practice time affected my confidence.” Institution B

#### 3.2.4 Theme 4: Looking Ahead

Students from both institutions provided constructive feedback on how simulation environments could be enhanced for better learning. They emphasized the need for more realistic mannequins, well‐maintained, and accessible simulation resources to maximize learning outcomes.

“The mannequin could be changed to a better one.” Institution A

“I would prefer having one with standard anatomy before the one with anatomical variations.” Institution A

“Improve the design; the teeth fall out, and it's hard to adjust fingers.” Institution B

“More mannequins are needed and better access to practice time.” Institution B

“Film stabilization could be improved, perhaps better holders or softer material for the mannequin's mouth.” Institution B

## 4 Discussion

This is the first study to explore students' perception of SBL in training for intraoral radiographic techniques, using a mixed‐methods approach across two institutions. The results showed that the students perceived SBL in oral radiology positively, with 85.86% of participants agreeing and strongly agreeing with all items in both institutions, along with an overall perception mean of 4.27. Remarkably, the students in Institution A were generally more positive than the students in Institution B, with statistically significant differences in the survey Items 1, 4, and 5. In contrast, no statistically significant differences were detected between male and female students. Interestingly, thematic analysis further reinforced the findings of the quantitative analysis, revealing the advantages of SBL, such as enhanced skill development, a safe learning environment, and bridging theory with practice. However, students also highlighted the barriers and limitations of SBL, including a lack of realism, technical issues, and limited access and resources. Finally, some actionable recommendations were suggested to improve the implementation of SBL in oral radiology education.

Overall, the combined quantitative results of the participating institutions in the current study were positive, suggesting that most students consider SBL as an appropriate method for teaching and learning intraoral radiography techniques. This is consistent with the findings of most studies conducted on students’ perceptions of a simulated dental learning environment [24, 25, 26, 27]. Out of the nine items in the survey, Items 7, 5, 8, and 9 had the highest positive mean in both institutions. This result supports that the simulated dental learning environment creates a psychologically safe learning space for novice learners and facilitates their smooth transition into clinical settings, as well as improves patient safety [28]. Of course, more practice reflects on increasing students' confidence levels in taking intraoral radiographs. By offering opportunities to practice without the pressure of real‐life clinical consequences, simulated settings help students build their confidence in mastering essential skills [6].

On the other hand, Item 3 received the lowest mean scores, followed by Item 2 at both institutions, which were related to the realism and translating radiographic concepts into practice, respectively. A notable drawback detected by the students was that the radiography mannequin did not accurately simulate the radiographic appearance of enamel, dentine, periodontal ligament space, and bone structure. It may be argued that this simulation is not essential for the acquisition of radiographic technique skills, and it has more impact on radiographic interpretation skills. While accurate simulation of natural teeth and supporting structures on radiographs seems ideal and may be achieved using high‐fidelity mannequins, it may also introduce unintended shortcomings. A previous study has reported [29] that increasing fidelity leads to an increase in the students’ cognitive load and may be a source of distraction. Moreover, using the high‐fidelity mannequin can contribute to the learners' feeling overconfident [30]. Given that students in simulated settings typically focus on image acquisition rather than interpretation, high‐fidelity simulation may not be essential. Instead, skills in radiographic reporting and interpretations may be better imparted by using simulated cases during case‐based discussions or small group workshops [11, 12, 31]. However, it remains important to ensure that the application of radiographic geometric principles is adequately addressed during using the mannequin‐based training. In addition, virtual reality could complement mannequin‐based SBL by providing additional opportunities for spatial visualization and geometric understanding [15, 16].

The results showed that Institution A is more optimistic about the mannequin radiography teaching than Institution B, especially in Items 1, 4, and 5. This could be related to the difference in the training between the two institutions. Institution A teaches periapical, focusing on paralleling technique, and bitewing radiography using receptor holders. Whereas, Institution B teaches periapical radiographs, focusing on the bisecting angle technique, using finger support, and bitewing radiographs using bitewing tapes. This is also confirmed by the thematic analysis, as the main difficulty experienced by most participants from Institution B is related to the adjustment of the mannequin finger to support the sensor. These differences highlight inconsistencies in the teaching of oral radiology in different institutions, as there is no global consensus on how these techniques should be taught in undergraduate dental programs. Although the paralleling technique is widely considered to be most acceptable for periapical radiography, the bisecting angle technique may be required due to anatomical restrictions, insufficient occlusal support, limited viewing conditions, limited mouth opening, or young children [32, 33].

Remarkably, thematic analysis provided in‐depth insights that supported the quantitative findings, reinforcing students' positive views of SBL in oral radiology. The first two themes, enhanced skill development and a safe learning environment, and bridging theory with practice, aligned with the students’ high response scores in Items 7, 5, 8, 9, 1, and 6. Both quantitative and qualitative data confirm how students valued the simulation environment as an opportunity to safely master radiographic skills before actual patient interactions. On the other hand, Theme 3 highlighted the barriers and challenges “Barriers,” such as a lack of realism compared to real‐patient experience, technical and operational difficulties with mannequins, and equipment and access constraints. These limitations are consistent with the lowest‐scoring items in the survey items 2 and 3. In addition, the qualitative findings explored in depth the underlying reasons for students' low satisfaction, contributing to a more comprehensive understanding of the students' perception. Lastly, the students recommended some suggestions for improvement, featuring students’ critiques, including calls for dependable and accessible training materials and simulations. These themes not only align with the survey results but also offer practical direction for enhancing SBL environments in dental education.

This study explores students’ perceptions of SBL in training for intraoral radiographic techniques using a mixed‐methods approach across two institutions, an area that has received limited prior investigation. It emphasizes not only participants’ quantitative performance but also students’ rich qualitative feedback about how SBL improves their confidence, motor skills, awareness of patient safety, and application of theoretical knowledge within a controlled environment. In addition, the results from thematic analysis also point out the need to broaden the resource complement for realism concerning the simulation tools provided. These findings strongly confirm the argument in support of experiential learning and highlight the need for more empirical, data‐driven educational research that employs validated performance metrics, standardized assessment tools, and outcome‐based evaluation frameworks to inform curriculum design and enhance teaching effectiveness in dental education. This study identifies the contribution of SBL in not only skill acquisition but also fostering desirable clinical practice behaviors and preparedness, emphasizing its role in contemporary educational curricula in dentistry. In addition, it highlights the variation in teaching basic radiographic techniques across institutions, emphasizing the need for standardization in teaching oral radiology.

Despite the strengths of the present study, some limitations need to be acknowledged. Of those, the sample size was small, which may limit the generalizability of the findings. In addition, the qualitative analysis was based on the open‐ended questions, which may have restricted the depth of insight into the students' perception. Future studies could enhance understanding by incorporating focus group discussions and involving a larger, more diverse sample across multiple institutions. Moreover, it may be helpful to triangulate the perceptions of the students with their performance in competency assessments in oral radiography.

## 5 Conclusions

Notwithstanding the limitations of this study, the findings indicate that students value SBL as an effective approach for teaching core competencies in oral radiology and may serve as a valuable precursor to clinical training. Further improvements in the learning experiences of the undergraduate students may be achieved through improving radiology mannequin design to make it more user‐friendly and provide students more opportunities to consolidate their radiography skills in simulated settings before clinical practice. Moreover, the observed variation in teaching basic oral radiographic techniques underscores the need for greater standardization in teaching oral radiology by adopting the paralleling technique as the baseline for preclinical instruction and developing unified instructor training guidelines.

## Funding

The authors have nothing to report.

## Conflicts of Interest

The authors declare no conflicts of interest.
